# Isoelectric Focusing Fractionation Method for Signal Enhancement in Detection of Inactivated Biological Agents Using Matrix‐Assisted Laser Desorption/Ionization Mass Spectrometry

**DOI:** 10.1002/elps.202400052

**Published:** 2025-01-02

**Authors:** Filip Duša, Jiří Šalplachta, Marie Horká, Kamila Lunerová, Veronika Čermáková, Michal Dřevínek, Oldřich Kubíček

**Affiliations:** ^1^ Institute of Analytical Chemistry of the Czech Academy of Sciences Brno Czech Republic; ^2^ National Institute for Nuclear, Chemical and Biological Protection Kamenna Czech Republic

**Keywords:** biological agents, chip, fractionation, isoelectric focusing, pathogenic bacteria, whole cell separation

## Abstract

Timely identification of highly pathogenic bacteria is crucial for efficient mitigation of the connected harmful health effects. Matrix‐assisted laser desorption/ionization time‐of‐flight mass spectrometry (MALDI‐TOF MS) of intact cells enables fast identification of the microorganisms based on their mass spectrometry protein fingerprint profiles. However, the MALDI‐TOF MS examination must be preceded by a time‐demanding cultivation of the native bacteria to isolate representative cell samples to obtain indicative fingerprints. Isoelectric focusing (IEF) is capable of separating bacterial cells according to their isoelectric point while effectively removing other non‐focusing compounds from sample matrix. In this work, we present a divergent‐flow IEF chip (DF‐IEF chip) fractionation as an alternative way for sample clean‐up and concentration of bacterial cells to prepare samples usable for following MALDI‐TOF MS analysis without the need of time‐demanding cultivation. By means of DF‐IEF chip method, we processed four species of highly pathogenic bacteria (*Bacillus anthracis*, *Brucella abortus*, *Burkholderia mallei*, and *Yersinia pestis*) inactivated with H_2_O_2_ vapors or by heat treatment at 62.5°C for 24 h. The DF‐IEF chip method continually separated and concentrated the inactivated bacterial cells for subsequent detection using MALDI‐TOF MS. The content of the inactivated bacteria in the DF‐IEF chip fractions was evaluated with the MS analysis, where inactivated *Y. pestis* was found to be the most efficiently focusing species. Sensitivity analysis showed limits as low as 2 × 10^5^ colony forming units per mL for inactivated *B. anthracis*.

**Abbreviations**
BSL‐3: biological safety level 3
CFU: colony forming units
DF‐IEF: divergent‐flow isoelectric focusing
LMM: low molecular‐mass
SA: 3,5‐dimethoxy‐4‐hydroxycinnamic acid

## 1 Introduction

Humans, animals, or plants are constantly in contact with various bacteria whether they are harmless, beneficial, or pathogenic. The identification of unknown bacteria has been one of the major challenges in microbiology as the rapid and accurate identification of possible dangerous pathogenic species is essential for effective dealing with the situation. Highly pathogenic species, including bacteria that may be causative agents of plaque or anthrax, are object of particular interest as they can be exploited for bioterrorism or biological warfare. Timely identification of such microorganisms is, therefore, high priority for effective mitigation or minimalization of the possible threat.

There are numerous methods used for detection and identification of the biological agents, such as culture‐based conventional methods, immunological detection methods, nucleic acid‐based assays, ligand‐based (aptamers and peptides) detection, and biosensors [1]. Especially conventional methods can be laborious and can take up to a few days to complete [2]. Nucleic acid–based methods are powerful techniques enabling precise identification of the bacteria; however, they need to be performed selectively which poses a problem in terms of time and economic demands, especially in the case of testing unknown samples. Proteomic approaches which started to develop over the last two decades can also provide fast and reliable identification of microorganisms [3]. Intact cell matrix‐assisted laser desorption/ionization time‐of‐flight mass spectrometry (MALDI‐TOF MS) has been suggested as a rapid and reliable technique for bacterial identification [4] with sensitivity as low as 10^3^ colony forming units (CFU) per mL [2]. This technique relies on comparison of the large databases of bacteria reference mass spectra [5] with the detected patterns of the microbial cell proteins. MALDI‐TOF MS has been used for identification of various microorganisms including *Bacillus anthracis* isolates [6] and spores in suspicious powders [7] as such moieties can be easily transported in sealed containers in low amounts which makes them hard to detect during security checks.

Working with highly pathogenic bacteria requires well‐trained personnel together with the high standard of personnel protection, including biological safety level 3 (BSL‐3) laboratories [1]. Methods enabling inactivation of the bacteria eliminate the potential threat and greatly facilitate the handling and analysis of the material [8, 9, 10, 11]. Mild inactivation using H_2_O_2_ vapors prevents the ability to reproduce but preserves the integrity of the cells [8]. The drawback of this approach is that the identification methods requiring bacterial growth will be effectively excluded after treatment.

Isoelectric focusing (IEF) is an electrophoretic method capable of separating ampholytes and bioparticles with ampholytic properties (e.g., bacteria and viruses) based on their isoelectric point (p*I*) [12, 13, 14, 15]. The analyte is desalted, and the concentrated sample matrix is removed during the IEF separation, which renders the method very suitable for separations of low concentrated and salty samples or extracts which are hard to analyze with other separation techniques.

Recently, we have shown a divergent‐flow IEF chip (DF‐IEF chip) capable of fractionation and concentration of bacterial species *Escherichia coli*, *Bacillus cereus*, *Pseudomonas aeruginosa*, and *Streptococcus agalactiae* inactivated by H_2_O_2_ vapors and their subsequent analysis using MALDI‐TOF MS without the need for prior cultivation samples [8]. This manuscript demonstrates application of the developed DF‐IEF chip method for model fractionation and concentration of four highly pathogenic bacterial species marked as risk group 3 biological agents—*B. anthracis*, *Yersinia pestis*, *Burkholderia mallei*, and *Brucella abortus*—inactivated using H_2_O_2_ vapors or by heat treatment to present an alternative way for preparation and clean‐up sample for following MALDI‐TOF MS analysis without the need of time‐demanding cultivation. Moreover, processing the inactivated material enables more simple handling in terms of biosafety precautions.

## 2 Methods and Chemicals

### 2.1 Chemicals

All chemicals used in the study were of analytical or higher grade. Sodium hydroxide, orthophosphoric acid, glycerol, patent blue V sodium salt, non‐ionogenic surfactant Brij 35, ethanol (EtOH), and polyethylene glycol (PEG 10 000, Mw 10 000) were purchased from Sigma‐Aldrich (Merck KGaA, St. Louis, MI, USA). Overall, 40% of biolyte 3/10 ampholytes were purchased from Bio‐Rad (Hercules, CA, USA). α‐Cyano‐4‐hydroxycinnamic acid was purchased from Bruker Daltonics (Billerica, Massachusetts, USA). Acetonitrile and 2‐propanol were purchased from VWR International (Randor, PE, USA). High‐resolution ampholyte, pH 2–4, ampholyte pH 3–4.5, 2‐morpholino‐ethanesulfonic acid monohydrate, 3‐morpholino‐propanesulfonic acid, *N*‐[tris‐(hydroxymethyl)‐methyl]‐3‐amino‐2‐hydroxy‐propanesulfonic acid and trifluoroacetic acid (TFA) were supplied by Fluka Chemie GmbH (Buchs, Switzerland). *N*‐(2‐Acetamido)‐2‐aminoethanesulfonic acid and 2‐[4‐(2‐hydroxyethyl)‐1‐piperazinyl]‐ethanesulfonic acid was obtained from Merck (Darmstadt, Germany). l‐Aspartic acid was from LOBA Chemie (Vienna, Austria). 3,5‐Dimethoxy‐4‐hydroxycinnamic acid (SA) and protein calibration mixture ProMix2 were purchased from LaserBio Labs (Sophia‐Antipolis Cedex, France). The in‐house developed low molecular‐mass (LMM) p*I* markers: 2.61, 3.93, 5.299, 7.24, and 9.78 were used for tracing the pH gradient [16, 17, 18, 19]. Deionized water was used for preparation and dilution of all solutions.

### 2.2 DF‐IEF Chip Running Solutions

Anolyte (100 mM H_3_PO_4_) along with catholyte (150 mM NaOH) were prepared by dilution of 85% H_3_PO_4_ and 1 M NaOH stock solutions. The input solution was composed of 1% (w/V) biolyte 3/10, 4 µg mL^−1^ LMM p*I* marker Patent blue V with p*I* 2.1 (green/blue), 32 µg mL^−1^ LMM p*I* marker 7.24 (yellow), and 10 µg mL^−1^ LMM p*I* marker 9.78 (violet). A total of 5 mL sample solution was prepared by adding 50 µL of the bacterial sample (i.e., glycerol dispersion) to 4950 µL of the input solution. Finally, a thorough degassing was applied to all solutions by simultaneous evacuation and ultrasonication for duration of 5 min.

### 2.3 Safety Considerations

The potentially pathogenic bacteria used in this work belong to the risk group 3 [20], and the work with the vital bacterial cultures was strictly contained within BSL‐3 laboratories. All experiments with inactivated bacteria were performed according to the instructions for the work with infective materials, and the use of protective equipment together with regular disinfection and decontamination of the laboratory equipment was maintained to avoid any contamination by microorganisms.

### 2.4 Microorganism Description, Cultivation, and Inactivation

Selected highly pathogenic bacteria *B. anthracis* vaccination strain *An* *traxen SU025*, *Y. pestis 570*, *B. mallei 12938T*, and *B. abortus 5660T* were obtained from National Institute for Nuclear, Chemical and Biological Protection (Kamenna, Czech Republic). All the selected bacteria belong to the risk group 3 biological agents according to the Annex III of the Directive 2000/54/EC of the European Commission [20]. *B. anthracis* vaccination strain *Antraxen SU025* and *B. mallei 12938T* were cultivated on tryptone soy agar (Oxoid Ltd., Basingstoke, UK) for 24 h on petri dish at 37°C, *Y. pestis 570* was cultivated on tryptone soy agar (Oxoid Ltd., Basingstoke, UK) on petri dish for 24 h at 30°C, and *B. abortus 5660T* was cultivated on Columbia blood agar (MkB Test, Slovakia) on petri dish for 24 h at 37°C. The fresh colonies were harvested and lyophilized using freeze‐dryer (Labconco Free Zone 1, Kansas City, MO, USA).

The highly pathogenic bacteria (e.g., BSL‐3 category) need to be completely inactivated before being handled at a lower safety level laboratory [21]. Inactivation of the bacterial samples was done by H_2_O_2_ vapors as described in our recent publication [8]. Briefly, lyophilized cultures were dispersed in glycerol, and 20 µL aliquots of the dispersion were pipetted into separate wells of a 12‐well cell culture plate. The cell culture plate was placed into a tempered box (50°C) with inner ventilation together with flat vessel containing 50% H_2_O_2_ covered with LDPE foil. The box was sealed, and H_2_O_2_ vapor at concentration of 1500 ppm was let to inactivate the bacteria for 120 min at 50°C ± 1°C.

The second and following sets of experiments included *Y. pestis 570* cultivated as described above and inactivated by heat treatment. The grown cultures were harvested and diluted in physiologic solution to the concentration of 10^9^ CFU (evaluated by densitometer Densi‐La‐Meter, PLIVA‐Lachema, a. s., Brno, Czech Republic). The dispersed cultures were inactivated at 62.5°C for 24 h in thermo‐shaker block bath (MSC‐100, LABtechnik, s. r. o., Brno, Czech Republic). The inactivation of *B. anthracis* strain *Antraxen SU025* sample used in all experiments was always performed using H_2_O_2_ method described above. The inactivation was confirmed by cultivation of the sample on corresponding medium and temperature for 24, 48, 96, and 168 h. All cultivations gave negative results. Confirmed by the test results, the completely inactivated bacteria were then used for further DF‐IEF chip fractionation.

### 2.5 Determination of CFU Concentration of Inactivated Bacteria

The sensitivity of the DF‐IEF chip method was evaluated using inactivated *B. anthracis Antraxen SU025* sample. First, the *B. anthracis* colonies of cells grown on Columbia blood agar were lyophilized and weighted to 0.4 mL of glycerol. The sample was then split into two equal volumes. The first half was inactivated using H_2_O_2_ vapors and used for the DF‐IEF experiments, and the second half containing vital cells was used for concentration analysis. A series of dilutions was used to determine the number of CFU of each dilution after 24 h of incubation on the medium at 37°C. The acquired CFU values were then adjusted to the original dispersion of inactivated cells.

### 2.6 Capillary Isoelectric Focusing (cIEF) of Bacteria

The cIEF was performed according to the method published earlier [22]. A volume of 500 mm long fused silica capillary (Agilent Technologies, Santa Clara, CA) with 100 µm inner diameter (ID) and 360 mm outer diameter (OD) was used for analysis. The effective length was 350 mm. A measurement of 20 kV applied on the cathodic side by high‐voltage unit Spellman CZE 1000 R (Plainview, NY) was used during the focusing as well as mobilization. Ampholyte mixtures with pH ranges of 3–10, 2.0–4.0, and 3.0–4.5 were mixed in the ratio of 1:2:5, respectively, and used for analyses. LMM p*I* markers with p*I*s 2.01, 2.61, 3.93, 5.299, and 7.24 at concentrations of 25 µg mL^−1^ were used to trace pH gradient. Detection at 280 nm was performed by UV–Vis detector LCD 2082 (Ecom, Prague, Czech Republic). A volume of 40 mmol l^−1^ sodium hydroxide and 100 mmol l^−1^ orthophosphoric acid, both with the addition of 5% (v/v) EtOH, 0.3% (w/v) Brij 35, and 0.3% (w/v) PEG 10 000, were used as the catholyte and the anolyte solutions, respectively. The capillaries were rinsed with 5 mL of anolyte and subsequently with 5 mL of catholyte before each cIEF run. The CFU concentration of the analyzed bacteria was adjusted to 1 × 10^7^ CFU mL^−1^, and the volume of 100 nL was injected using hydrodynamic injection.

### 2.7 DF‐IEF Chip Fractionation

**FIGURE 1:**
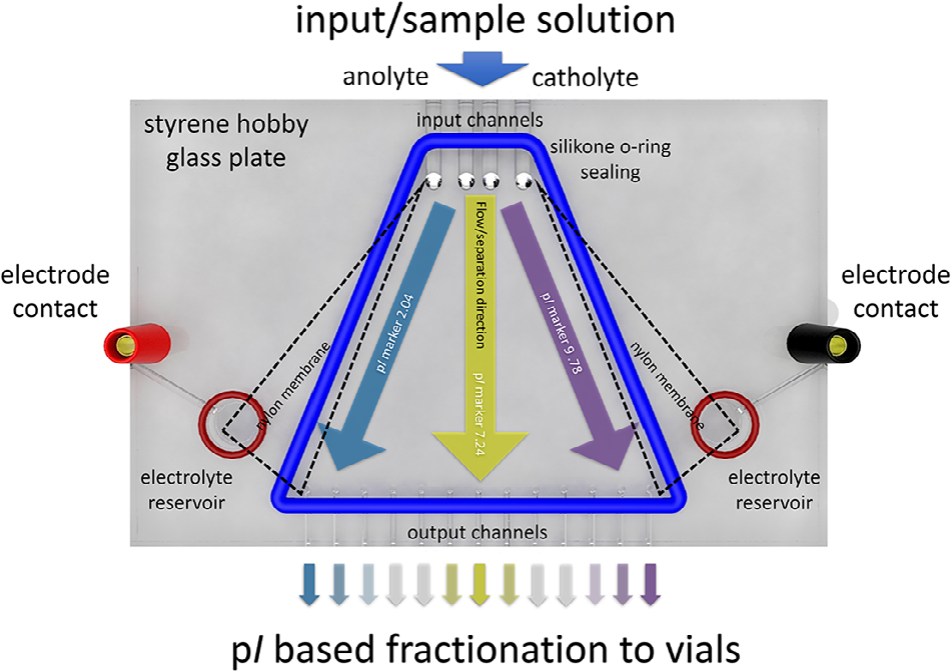
Scheme of DF‐IEF chip.

Construction of the DF‐IEF chip composed of two planar sheets of poly‐styrene hobby glass (thickness 8 and 5 mm for bottom and top sheet) along with its working principle was described in detail in our previous study [8]. The separation space features 32 mm width at the input side and 102 mm width at the output side, and the depth is linearly increasing by 0.4 mm from output to input side. The chip features 4 input channels and 13 collecting outputs (see Figure 1) connected to polytetrafluoroethylene (PTFE) tubing 1/16″ OD, 0.25 mm ID (Bohlender GmbH, Grünsfeld, Germany). The inputs are connected to Tygon LMT 55, 3‐stop tubing (Ismatec, Wertheim, Germany) with IDs of 0.38 mm (anolyte and catholyte) and 0.76 mm for sample solution. Reglo‐CPF Digital peristaltic pump (Ismatec) was used for delivery of the running solutions. During analysis, flow of 250 µL min^−1^ was maintained for sample solution and 50 µL min^−1^ for the anolyte and catholyte. Prior to the fractionation, the DF‐IEF chip was rinsed by running solutions to exchange the deionized water used for flooding of the chip. Subsequently, the electric field was applied by power supply Consort EV261 (Consort bvba, Turnhout, Belgium) using programmed linear increase of voltage from 0 to 500 V in 60 min with the limitation of 4 mA and 3 W. After the initial increase, the voltage was maintained at 500 V until the end of fractionation. The quality of the created pH gradient was visually monitored during the initial stage of increasing voltage by the inclusion of the p*I* markers in the input solution. Creation of the stable divergent lines of green, yellow, and violet p*I* marker stretched evenly over the separation space signalized stable pH gradient. The volume of 5 mL of the sample solution containing bacteria was then applied and, after 5 min, required to exchange the original input solution with the sample solution, a holder with 13 2.0‐mL micro test tubes was placed under the output tubing to collect the separated fractions. The collection was stopped after 10 min, and the fractions were sealed and kept at 6°C prior to the further analysis.

Even though the bacterial samples were completely inactivated, safety measures were applied to minimize any possible risks. Following the end of fractionation, the chip was rinsed with deionized water, and subsequently, 50% 2‐propanol was used for decontamination of the device. The decontaminating solutions were emptied from the chip by pumping air into the device, and the device was disassembled, manually rinsed with 100% 2‐propanol, and left to dry. All solutions introduced during or after the bacterial sample fractionation were disposed of as biohazard waste. Finally, laminar flow cabinet Aura Mini (BioAir, Siziano, Italy) was irradiated by a detachable UVC lamp for 15 min to sanitize the working area.

### 2.8 MALDI‐TOF MS of the DF‐IEF Chip Fractions

For creation of reference MS spectra of the investigated inactivated bacteria, 10 µL of 10 times diluted original inactivated bacterial dispersion in glycerol was mixed with 30 µL of SA solution (20 mg mL^−1^ in ACN/2% TFA, 3:2, v/v). The mixture was briefly vortexed and centrifuged, and the resulting supernatant (0.7 µL) was deposited on a MALDI sample plate. The DF‐IEF fractions were centrifuged at 14 000 *g* for 20 min and 0.7 µL of the supernatant, carefully taken at the bottom of the micro‐test tube right above the pellet of cells, was deposited on the MALDI sample plate. Modified sandwich deposition method was used for all the samples. Sample spots on the MALDI plate were first overlaid with 0.6 µL of SA solution and let dry at room temperature. Then, 0.7 µL of the sample was added onto the matrix layer. Finally, another layer of SA matrix (0.5 µL) was added onto the dried sample spots. All the bacterial samples were spotted in duplicates on the sample plate.

MS analyses were performed on an AB Sciex TOF/TOF 5800 System in the linear positive ion mode. Each mass spectrum was acquired by accumulation of a total of 2000 laser shots (40 different points randomly selected within sample spot by an acquisition software, 50 laser shots per point). Mass spectra were calibrated externally, and the data were processed using Data Explorer (ver. 4.8, AB Sciex).

## 3 Results and Discussion

### 3.1 cIEF of Inactivated Bacteria

Prior to the DF‐IEF fractionation, we estimated the fraction supposed to contain the inactivated bacteria cells according to their isoelectric point p*I*. We have shown that the inactivation changes the surface properties of the bacterial cells and, therefore, affects their p*I* [8]. Although it was not possible to analyze the native biological agents with cIEF, only the p*I* values of biological agents inactivated using H_2_O_2_ vapors were measured. The obtained p*I* values for the individual strains of species are shown in Table 1. Apart from inactivated *Y. pestis*, all other inactivated bacteria manifested a single main peak in cIEF.

**TABLE 1:** Isoelectric point (p*I*s) of the investigated inactivated bacteria determined by capillary isoelectric focusing (cIEF) analysis.

Inactivated bacteria strain	cIEF p*I*
*Yersinia pestis* 570	5.6/2.7
*Burkholderia mallei 12938T*	4.9
*Brucella abortus 5660T*	5.7
*Bacillus anthracis (Antraxen SU025)*	3.9

### 3.2 Analysis of Four Inactivated Pathogenic Bacterial Species Using DF‐IEF Chip

The optimization of the DF‐IEF chip operation was described in the earlier work [8]. Therefore, we proceeded directly to the DF‐IEF analysis. The inactivated bacteria were analyzed in the same top‐down order as shown in Table 1. The sample solution was made by mixing 20 µL of inactivated *Y. pestis*, *B. mallei*, or *B. abortus* glycerol dispersion with 4980 µL of input solution. In the case of *B. anthracis*, 50 µL of glycerol dispersion was mixed with 4950 µL of input solution. Dilutions were selected in regard to the available amounts of the inactivated bacteria. A volume of 10 µL aliquots of all glycerol dispersions were kept as reference for the MALDI‐TOF MS profiling (see Figure S1).

**FIGURE 2:**
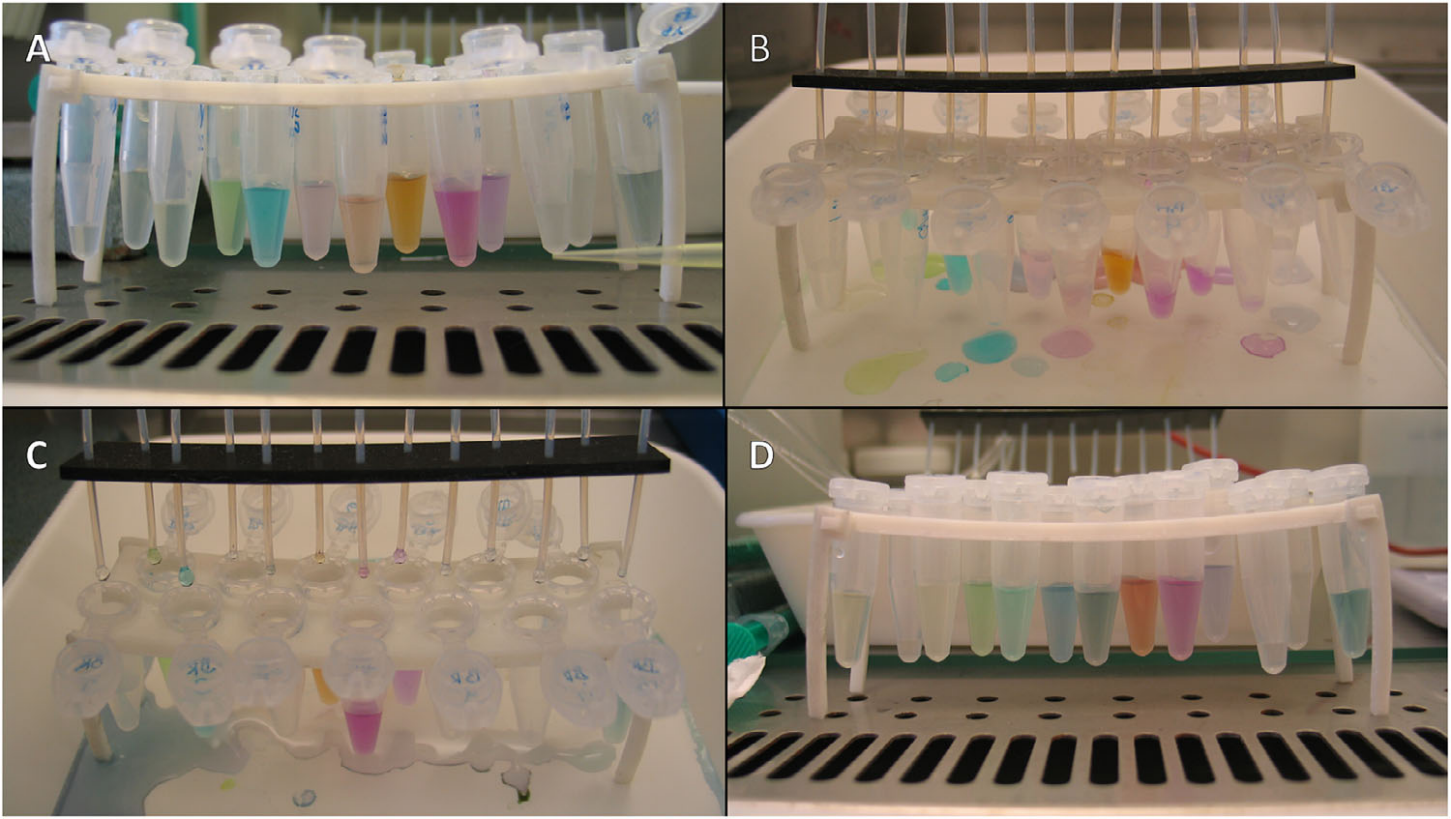
LMM colored p*I* markers focused and collected in the DF‐IEF chip fractions of inactivated bacteria: (A) *Yersinia pestis*, (B) *Burkholderia mallei*, (C) *Brucella abortus*, and (D) *Bacillus anthracis*.

Addition of the three p*I* markers of p*I* 2.1 (green/blue), 7.24 (yellow), and 9.78 (violet) enabled direct tracking of the pH gradient formation. Moreover, as the markers also accumulated in the collected fractions, the pH of the fractions could be determined (see Figure 2). The pH value of the fraction containing the major portion of each marker was assigned to its p*I*, and the pH values of the fractions in between were linearly interpolated with precision of one‐tenth of pH unit (see Table 2).

**TABLE 2:** The obtained fractions isoelectric point (p*I*s) and the detected presence of the inactivated bacteria.

Inactivated bacteria	Fraction												
	**1**	**2**	**3**	**4**	**5**	**6**	**7**	**8**	**9**	**10**	**11**	**12**	**13**
***Yersinia pestis***	A	A	A	2.1	**3.4**	4.7*	5.9*	7.2*	8.5	9.8	B	B	B
***Bacillus anthracis***	**A**	**A**	**A**	**2.1**	3.8*	5.5*	7.2*	**8.5**	9.8	B	B	B	B
***Brucella abortus***	A	2.1	**3.4**	**4.7**	5.9*	7.2*	8.5	9.8	x	B	x	B	B
***Burkholderia mallei***	A	2.1	x	**3.1**	**4.1**	5.2*	6.2*	7.2*	8.5	9.8	B	B	x

*Note*: The values of p*I*s were assigned to the fractions based on the linear interpolation of the p*I* markers positions (A = acidic, B = basic). Bold font highlights the fractions in which the bacteria were detected by MS analysis, and underlined fractions show the most concentrated samples with the highest MS spectra quality. Asterisk represent the fractions where the visible clusters were focused. Clogged fractions marked by “x” were skipped from pH interpolation.

During the course of analysis, violet clusters could be observed near the output of the separation space with all tested bacteria. The clusters eventually formed a focused stream which was collected through the output tubing as described in Table 2. The results suggest that a portion of bacterial cells aggregated at pH approaching their p*I*s, and possibly the violet p*I* marker was adsorbed on these cell clusters thus highlighting them. Although the fractions with observed cluster did not completely overlap with the MALDI‐TOF MS analysis, they were at least partly indicative of where the bacteria focused.

### 3.3 Mass Spectrometry Analysis of DF‐IEF Fractions

The MALDI‐TOF MS analysis of all collected fractions revealed that the inactivated bacteria focused in rather broad peak‐profiled zones. The profiles corresponding to the previously measured reference samples of inactivated bacteria were found in multiple fractions in acidic region (see Table 2 for the “bold font” emphasized fractions). As the focusing efficiency together with MALDI‐TOF MS identification are significantly dependent of the analyzed species [21], we discuss each analyzed bacterial species separately on the following lines.

**FIGURE 3:**
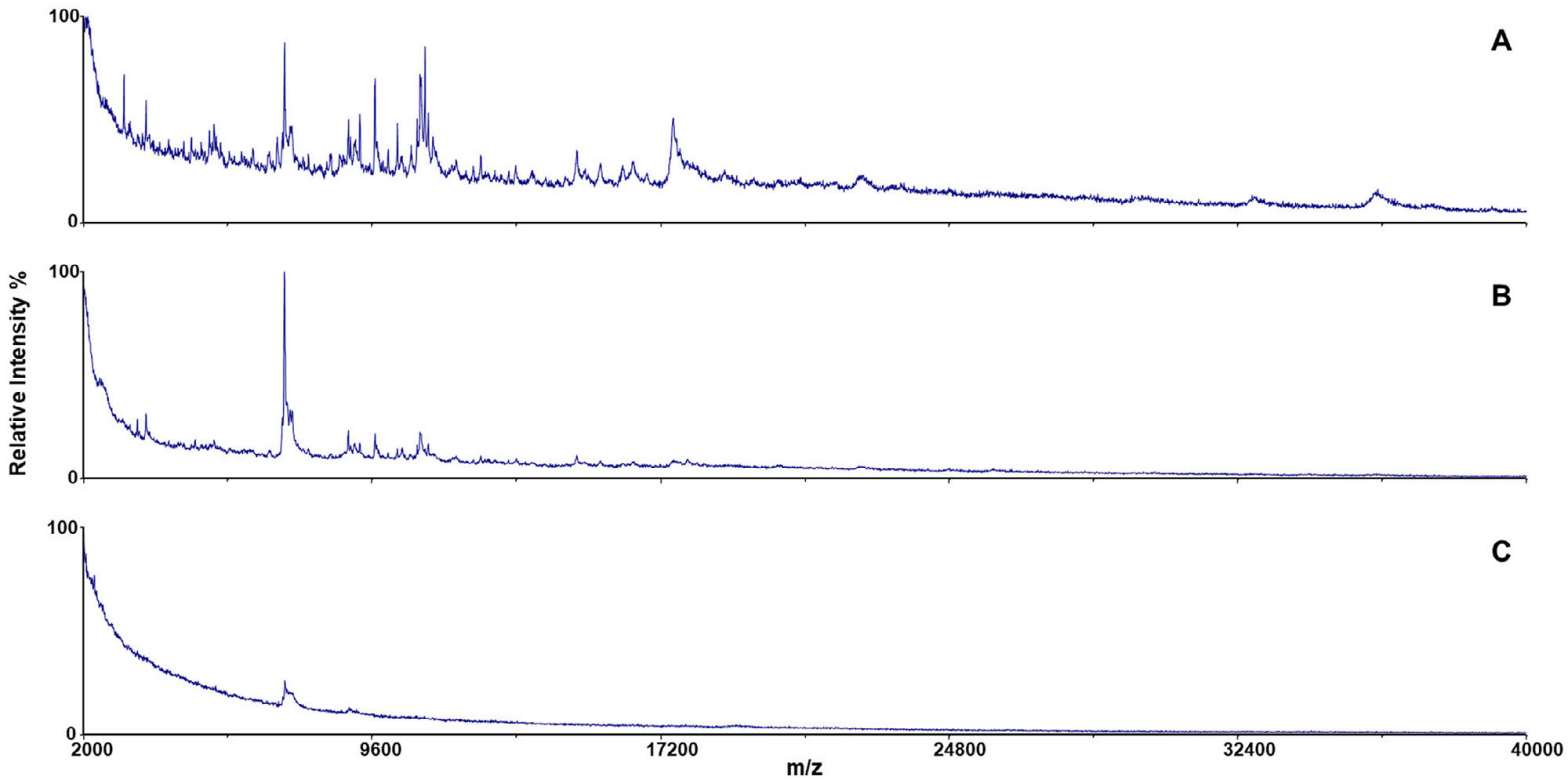
MALDI‐TOF mass spectra of H_2_O_2_ inactivated *Yersinia pestis* in the analyzed DF‐IEF chip fractions: (A) fraction nr. 5 (pH 3.4); (B) fraction nr 6. (pH 4.7, the main fraction); (C) fraction nr. 7 (pH 5.9).

The most efficiently focusing bacterium species was the inactivated *Y. pestis*. It focused on three adjacent fractions, nr. 5, 6, and 7. The middle fraction nr. 6 with pH 4.7 showed the most intensive peaks, and thus, it was marked as the main fraction (Figure 3). Two adjacent fraction nr. 5 (pH 3.4) and nr. 7 (pH 5.9) showed lower intensity, suggesting peak profile of the focused zone of the bacterium. The pH of the main fraction was close to the p*I* determined with cIEF. The MS profile of the fraction nr. 4 (pH 2.1) with very low signal intensity suggests that the second p*I* determined by cIEF (2.7) was probably not relevant to the inactivated cells (see Table 1). Clots were observed to focus in the fraction nr. 6 (pH 4.7) and nr. 7 (pH 5.9) closely correlating with the observed reference MS signals of the inactivated bacterium. Two further repetitions with heat‐inactivated *Y. pestis* were performed. The bacterial cells were detected by MS in the fraction nr. 4 (pH 3.6), nr. 5 (pH 5.1, main fraction), and nr. 6 (pH 6.5) in the second run and in the fraction nr. 5 (pH 3.4), nr. 6 (pH 4.7, main fraction), and nr. 7 (pH 5.9). This shows very stable and repeatable separation as shown previously [8]. It should be noted that a slightly different MS profile was obtained from heat‐inactivated *Y. pestis*; see Figure S2 for comparison.

**FIGURE 4:**
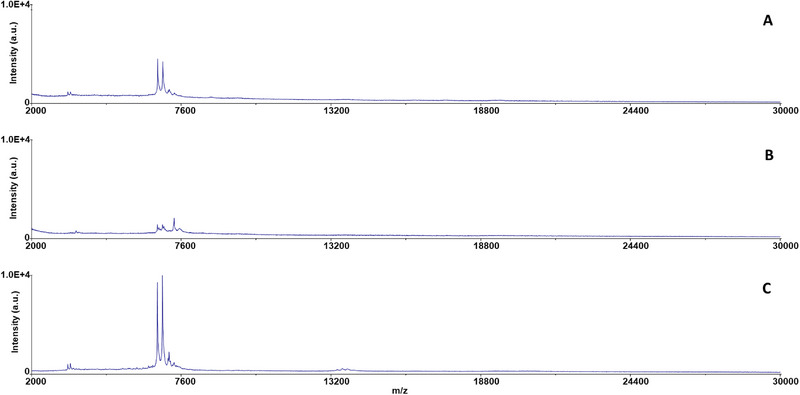
MALDI‐TOF mass spectra of inactivated *Bacillus anthracis* samples: (A) reference spectra of inactivated *B. anthracis* cells in glycerol dispersion (concentration 2.5 × 10^7^ CFU mL^−1^); (B) input solution imitating a “real” diluted inactivated sample (concentration 2.0 × 10^5^ CFU mL^−1^); (C) DF‐IEF fraction nr. 5 (pH 3.8, the main fraction). The absolute intensity of 10 000 a.u. (the *y* axis) was set in all three sub‐spectra to demonstrate the improvement and enhancement of mass spectrum of the inactivated bacterial sample obtained after DF‐IEF fractionation.

The benefit of the DF‐IEF chip method is illustrated in Figure 4 showing the signal enhancement of the inactivated *B. anthracis* sample, comparing the MS spectra of input solution containing inactivated cells imitating a “real” diluted sample (concentration 2.0 × 10^5^ CFU mL^−1^) versus the MS spectra of the output fraction after DF‐IEF separation. Figure 4A shows a reference MS spectrum of *B. anthracis* inactivated using H_2_O_2_ in glycerol dispersion (after dilution as described in article 2.8), and Figure 4B demonstrates the MS spectra of the input solution, and Figure 4C shows the main fraction nr. 5 (pH 3.8). However, the focusing of inactivated *B. anthracis* showed the lowest efficiency within the whole series of the bacteria species tested. *B. anthracis* was detected in fraction nrs. 1–8 after the DF‐IEF chip analysis, including all three acidic fractions below the pH 2.1. The most basic fraction, where the bacterium was detected, was fraction nr. 8 (pH 8.5). The fraction nr. 4 (pH 2.1), nr. 5 (pH 3.8), and nr. 6 (pH 5.5) were identified as the main fractions, and the most intensive MS profile was found in the fraction nr. 5 (pH 3.8). This correlates very well with the p*I* 3.9 determined by cIEF (see Table 1). This could be partly due to the simple profile and thus easy detection of the *B. anthracis* (see Figure 4). Moreover, sporulation of *B. anthracis* might be another reason why the bacterium was found in so many fractions. Transition to spore form with connected formation of poly‐γ‐d‐glutamate capsule significantly changes the composition of the cell surface, and the negatively charged polymer lowers the p*I* of the cell [23]. The H_2_O_2_ vapors inactivation may also affect the permeability of the cell wall and the associated value of p*I* for some bacteria [8].

**FIGURE 5:**
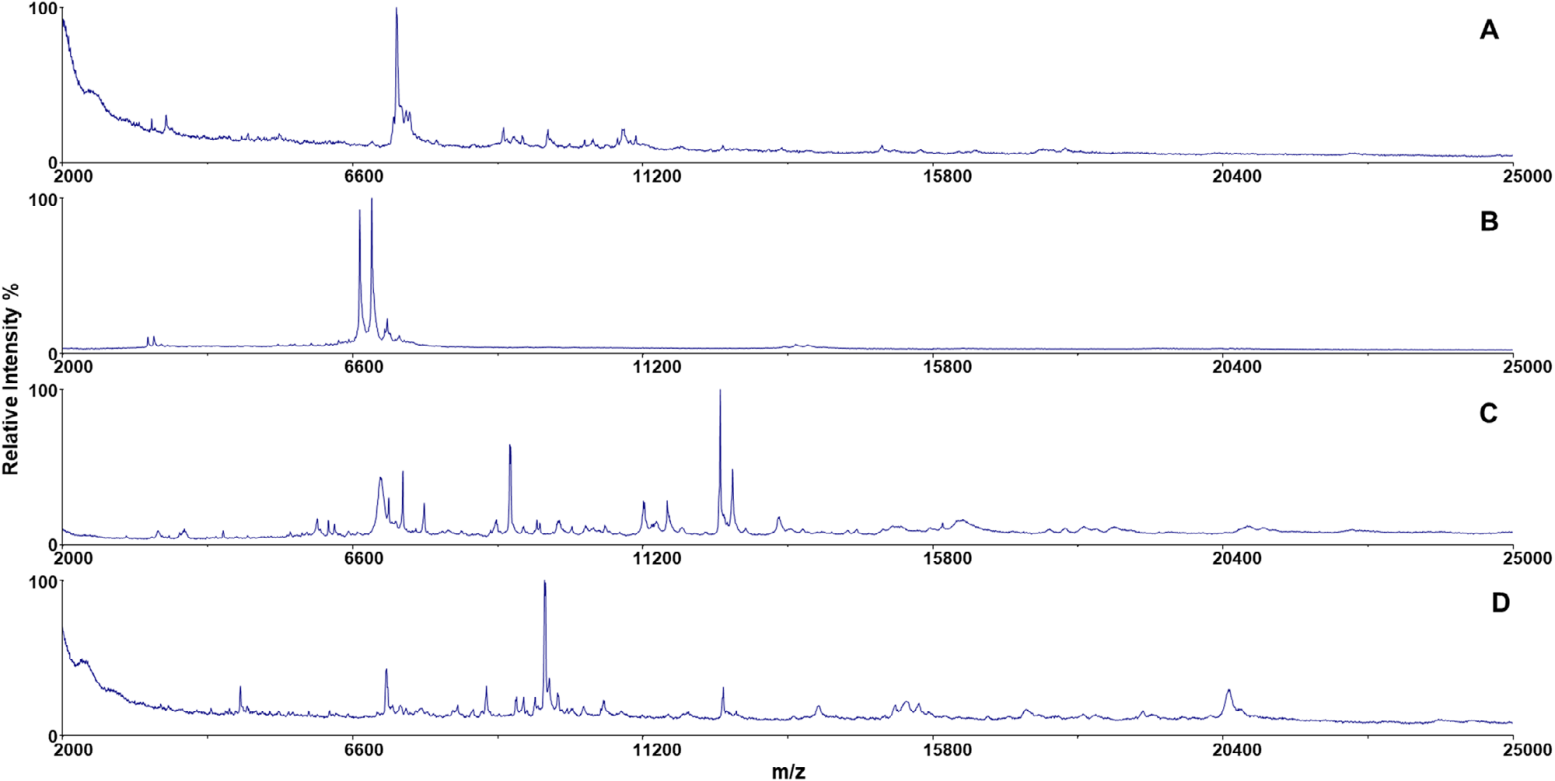
MALDI‐TOF mass spectra of the main fractions of the investigated inactivated bacteria: (A) *Yersinia pestis* (fr. 6); (B) *Bacillus anthracis* (fr. 5); (C) *Brucella abortus* (fr. 4); (D) *Burkholderia mallei* (fr. 7).

Inactivated *B. abortus* was also focused in three fractions. The bacterial MS profile (Figure 5C) detected in fraction nr. 3 (pH 3.4), nr. 4 (pH 4.7), and nr. 6 (pH 7.2) with the first two having the most quality spectra. Fraction nr. 5 (pH 5.9) did not show the bacterial presence. The possible reason may be the effect of the observed clusters focusing primarily around the pH 5.9 corresponding to bacterium p*I* of 5.7 (see Table 1) and then clogging the stream of the fraction nr. 5 and redirecting the focused bacterial cells to adjacent outlets nrs. 4 and 6 (see Table 2). As the outcome, a very little volume of the fraction nr. 5 was collected. This was unexpected, and the reason and effect of the clot formation need to be further studied in more detail. However, due to the limited amount of inactivated pathogenic material, this will be done in future studies.

Inactivated *B. mallei* was not well focused, and it was detected in several fraction nrs. 4, 5, 6, 7, and 8 in the pH region from 3.1 to 7.2. Fraction nr. 5 (pH 4.1) and nr. 7 (pH 6.2) showed the best identification MS profiles and were selected as the main fractions. The aggregating clusters were observed in the three fraction nrs. 6, 7, and 8 from pH 5.2 to 7.2 The p*I* of 4.9 of *B. mallei* determined using cIEF lay approximately in the middle of the interval of all fractions, where the bacterium was identified. This suggests that inactivated *B. mallei* focused accurately, but with rather low efficiency. Further optimization of the DF‐IEF conditions is required to improve the efficiency of fractionation of this particular species.

Mass spectra of the main fractions of all four inactivated bacteria are displayed in Figure 5 showing the characteristic mass profiles of the studied inactivated bacteria corresponding to previously measured reference MS spectra of the inactivated bacteria.

### 3.4 Sensitivity of the DF‐IEF Chip for Inactivated Pathogenic Bacteria

The bacteria *B. anthracis Antraxen SU025* inactivated using H_2_O_2_ vapor in glycerol dispersion was selected as the model organism for the sensitivity testing due to the simple MS profile as described in the previous section. The original glycerol dispersion contained 2.5 × 10^7^ CFU mL^−1^. A total of 49 mg (38.9 µL) of the glycerol dispersion was diluted into 4950 µL in the input solution resulting in a concentration of 2.0 × 10^5^ CFU mL^−1^. Two further glycerol dispersion samples with lower concentrations were prepared by successive dilutions of the original glycerol dispersion by water. This resulted in the concentrations of 2.0 × 10^4^ and 2.0 × 10^3^ CFU mL^−1^ in the sampled input solution. Characteristic MALDI‐TOF MS signal of inactivated *B. anthracis* was detected with the original glycerol dispersion and the DF‐IEF chip fraction nr. 5 from fractionation of the input solution containing 2.0 × 10^5^ CFU mL^−1^. All other determinations featured either low‐quality spectra with low peaks and high noise or no signal at the expected masses (see Figure 4, Table 3, and Figure S3).

**TABLE 3:** Inactivated *Bacillus anthracis* divergent‐flow isoelectric focusing (DF‐IEF) sample concentrations in connection to quality of the identification profiles.

Inactivated *Bacillus anthracis* CFU concentration vs. MS identification				
Glycerol dispersion (CFU mL^−1^)	MS identification	DF‐IEF input solution (CFU mL^−1^)	MS identification	MS identification in DF‐IEF chip fractions
2.5 × 10^7^	Yes	2.0 × 10^5^	No^a^	Yes
2.5 × 10^6^	No^a^	2.0 × 10^4^	No	No^a^
2.5 × 10^5^	No	2.5 × 10^3^	No	No^b^

^a^Weak and noisy signal not usable for identification.

^b^Only traces of the signal observed.

The collected volume of the fractions reached 0.5 mL which corresponds to 1 × 10^5^ CFU in fraction (2.0 × 10^5^ CFU mL^−1^ in the input solution). IEF application could increase the CFU concentration up to a factor of 11 (number of fractions minus border fractions with anolyte and catholyte). Furthermore, the identification sensitivity was also increased by removing matrix compounds present in the original sample. Ampholytes present in each fraction did not show high effect on the resulting mass spectra. The increase of the signal can be directly seen by comparing Figure 4B before and Figure 4C after the DF‐IEF fractionation. It is also confirmed in Table 3, which shows the lowest concentration for the *B. anthracis* identification. Our findings are in agreement with the MALDI‐TOF MS sensitivity limits suggested in the literature [2]. Furthermore, the measured sensitivity limit was still one order of magnitude lower in comparison to the data obtained for *E. coli* (2.1 × 10^6^ CFU mL^−1^) using the same instrumentation [8]. Considering the continuous nature of the DF‐IEF chip separation, the sensitivity could be further increased by using longer collection times, provided the surplus of input solution with a biological agent.

### 3.5 Benefits and Limitations of DF‐IEF Sample Fractionation for MALDI‐TOF MS Analysis

The main theme of this manuscript is the demonstration of the DF‐IEF fractionation benefits for the clean‐up and concentration of the biological pathogens as an alternative way for sample preparation for the following analysis by MALDI‐TOF MS technique which usually requires time‐demanding cultivation and handling of the vital cells connected with demanding biosafety measures.

Recently, liquid chromatography coupled to MS was used to analyze an extensive group of pathogenic bacteria (over 50 strains of different species) [24], including the species studied in this work. However, the authors used a different approach where the bacteria were first extracted from the sample matrix (e.g., culture media), lyzed, and subsequently digested using trypsin. Therefore, the samples required a few hours of preparation prior to the analysis by liquid chromatography. DF‐IEF chip fractionation requires much less handling and chemicals while being able to deliver cell samples usable for following MALDI‐TOF MS analysis. DF‐IEF chip is also able to fractionate inactivated cells with lower biosafety demands for the personnel and enables MALDI‐TOF MS analysis of non‐vital cells. Moreover, DF‐IEF fractionation is an option for samples where the cultivation is for some reason not possible.

The usual process for identification of biological agents by means of commercial MALDI‐TOF MS databases (e.g., MALDI BioTyper system) includes cultivation of the vital cells and subsequent MALDI‐TOF MS analysis of the MS spectra of protein fingerprints. Although the protein profile is characteristic for each genus and species, it also fluctuates depending on cultivation and/or inactivation conditions. For this reason, we prepared our own reference MS spectra database covering the investigated inactivated bacterial species using the glycerol dispersions of cells as the reference (see Figure S1).

To test applicability of the current commercial databases for inactivated bacteria, the fractionated bacteria samples (heat‐inactivated *Y. pestis* and H_2_O_2_‐inactivated *B. anthracis*) were analyzed by commercial identification MALDI‐MS protocol, and the obtained spectra were evaluated using the MALDI BioTyper Security‐Relevant (SR) Library (see Supporting Information for further details). Not surprisingly, the MALDI BioTyper log score values of fractions of both bacteria were below 1.7 meaning that the identifications were considered unreliable to the genus and species. Figure S4 shows the difference between mass spectra of native (database based) and inactivated *B. anthracis* (measured). This further supports the future need of a specific identification database for inactivated bacteria.

## 4 Concluding Remarks

MS of whole cells is a powerful tool for fast identification of bacteria. However, its sensitivity greatly depends on the sample matrix. We have presented a novel method for how to fractionate bacteria prior to their analysis using MALDI‐TOF MS without the need of time‐demanding cultivation. In DF‐IEF fractionation, a few mechanisms work together to provide concentrated fractions of bacterial cells. First, the bacterial cells are separated in vertical divergent flow by horizontally applied electric field to the fractions with pH respective to their p*I*. Second, salts and charged low‐molecular‐mass contaminants are removed during the focusing process. Third, the DF‐IEF is applicable for inactivated bacteria which makes the sample handling simpler regarding the biosafety measures. Importantly, the complete system (i.e., the parts in the contact with sample) can be decontaminated using isopropanol or other bactericide liquid mixtures. This makes it an applicable tool for purification of bacterial cells, including the inactivated highly pathogenic species exploitable as biological agents for bioterrorism. It is an alternative time‐saving way for the preparation of samples for MALDI‐TOF MS analysis in situations when it is critically important to have broad palette of tools capable of resolving and identifying these biological agents in a short timeframe.

## Conflicts of Interest

The authors declare no conflicts of interest.

## Supporting information



Supporting Information

## Data Availability

The data that support the findings of this study are available from the corresponding author upon reasonable request.
