# Supplementary material for: Isoelectric Focusing Fractionation Method for Signal Enhancement in Detection of Inactivated Biological Agents Using Matrix‐Assisted Laser Desorption/Ionization Mass Spectrometry
Source: Electrophoresis. 2025 Jan 2;46(3-4):212–20. doi: 10.1002/elps.202400052 (PMC11865691; doi:10.1002/elps.202400052)
Supplement: Supplementary file 1 — Supporting Information [file ELPS-46--s001.docx]

**Supporting information**

Isoelectric focusing fractionation method for signal enhancement in detection of inactivated biological agents using matrix-assisted laser desorption/ionization mass spectrometry

Filip Duša ^1*^, Jiří Šalplachta ^1^, Marie Horká ^1^, Kamila Lunerová ^2^, Veronika Čermáková ^2^, Michal Dřevínek ^2^, Oldřich Kubíček ^2^

^1^ Institute of Analytical Chemistry of the Czech Academy of Sciences, Brno, Czech Republic

^2^ National Institute for Nuclear, Chemical and Biological Protection, Kamenna, Czech Republic

Figure S1


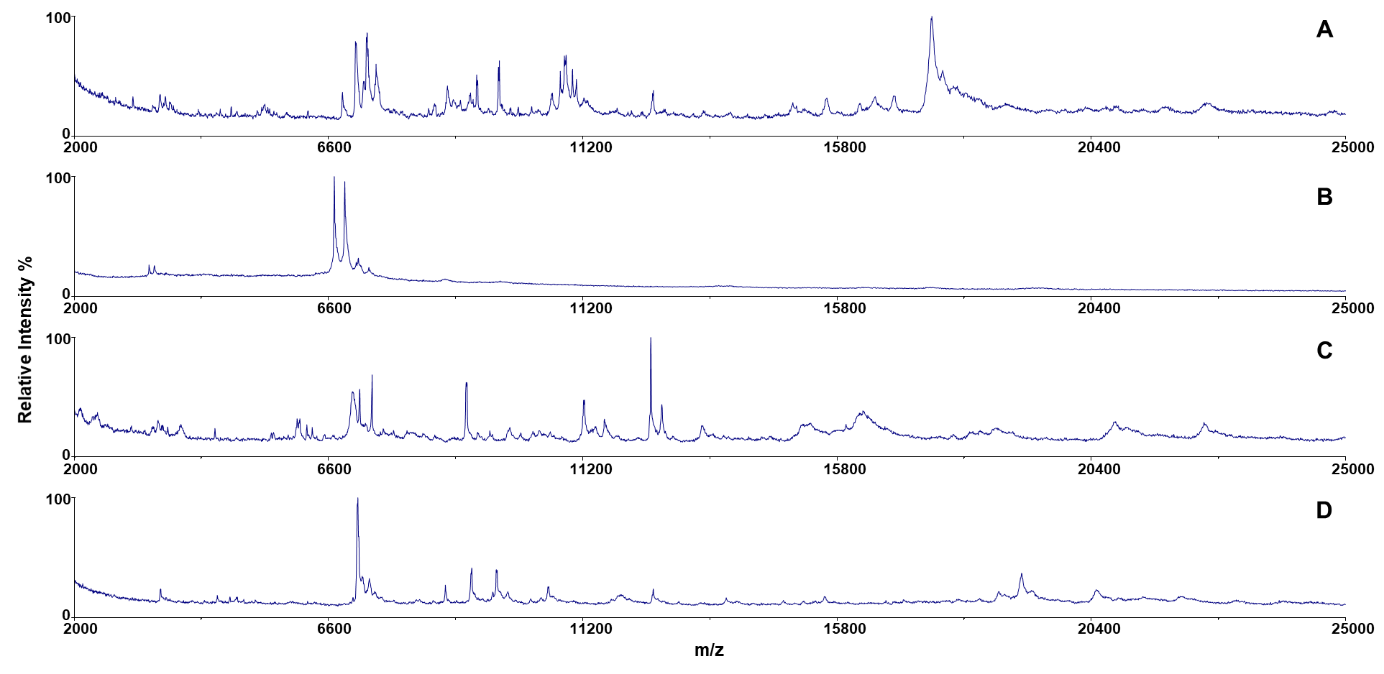


Figure S1 – Reference MALDI-TOF mass spectra of inactivated bacterial cells in glycerol dispersion. A – *Yersinia pestis*; B – *Bacillus anthracis*; C – *Brucella abortus*; D – *Burkholderia mallei*.

Figure S2


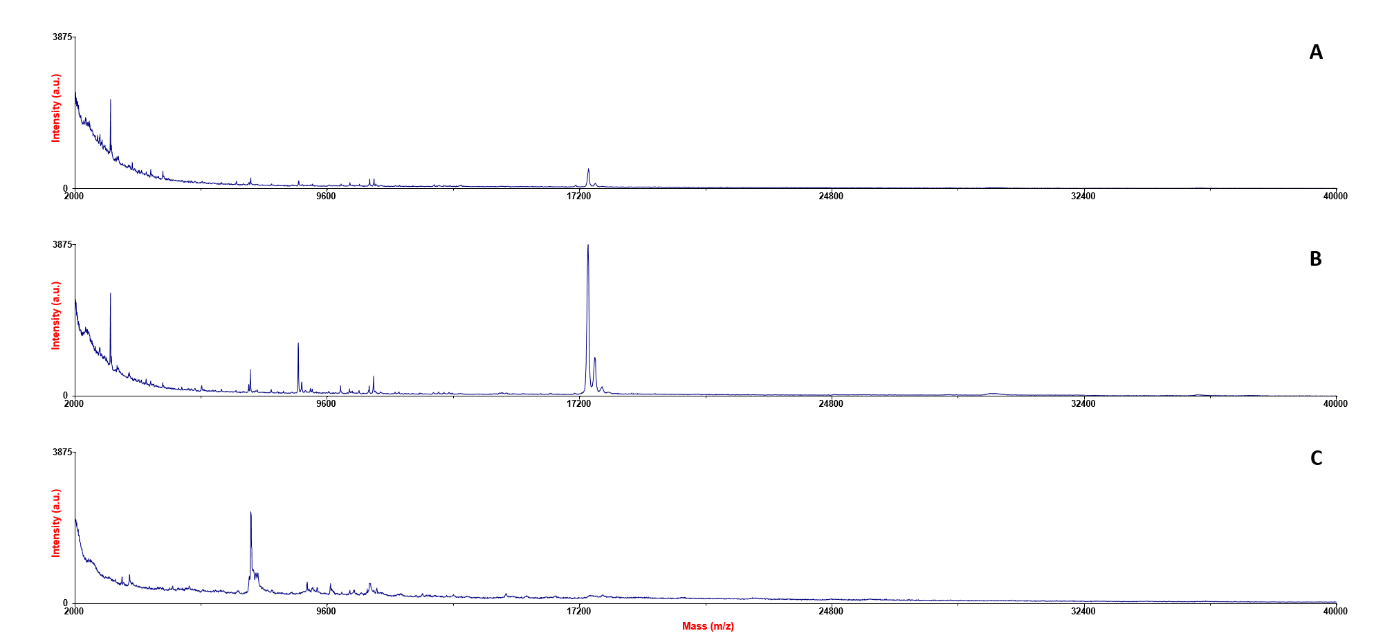


Figure S2 - Comparison of MALDI-TOF mass spectra of *Yersinia pestis* samples inactivated by two different methods. A – input sample used for DF-IEF chip fractionation (heat-inactivated); B – main fraction of *Yersinia pestis* (heat-inactivated); C – main fraction of *Yersinia pestis* (H_2_O_2_-inactivated).

Figure S3


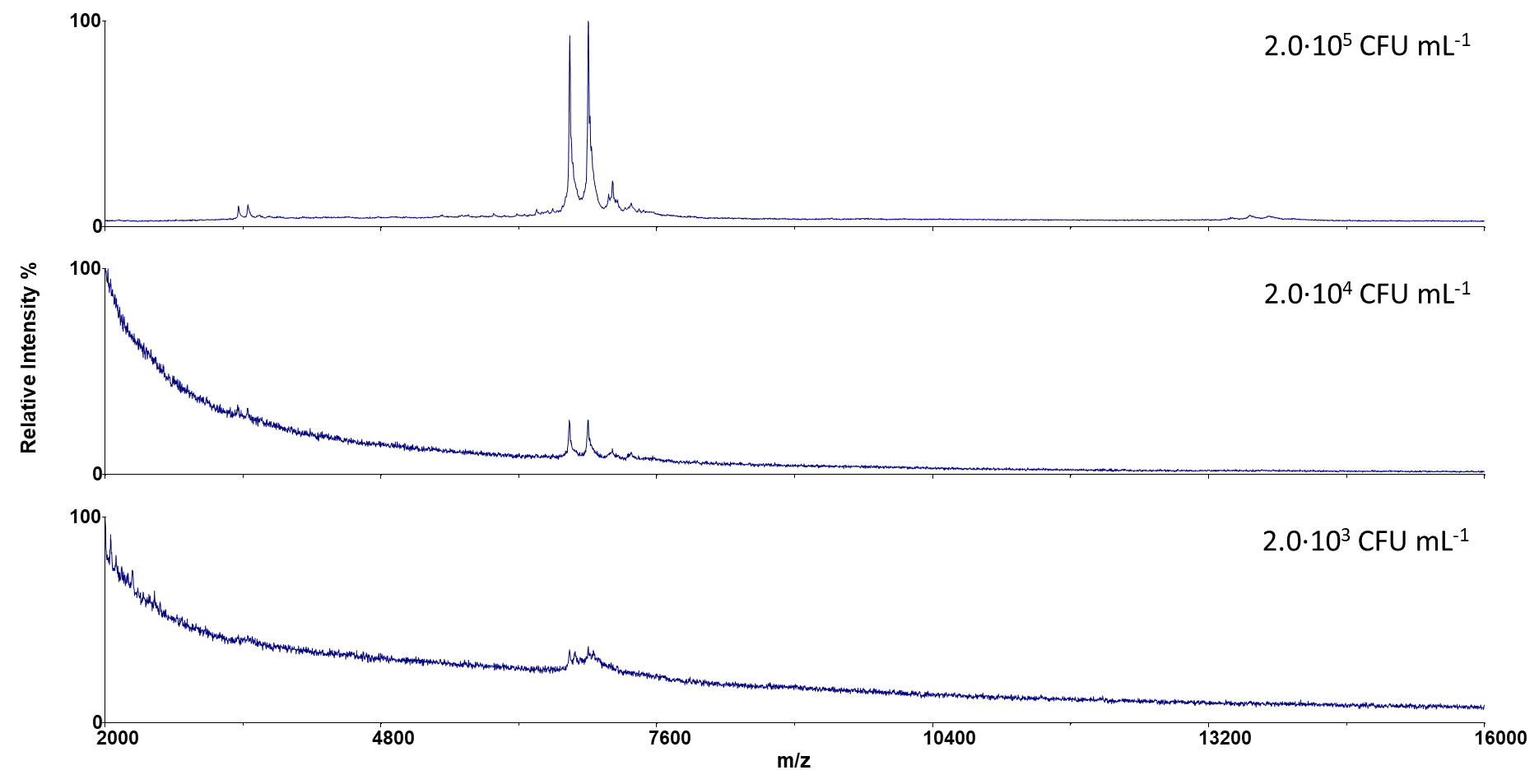


Figure S3 – MALDI-TOF mass spectra of inactivated *Bacillus anthracis* samples from sensitivity analysis (three different cell concentrations) acquired after DF-IEF fractionation from fraction nr. 5. For concentrations 2.0·10^-4^ and 2.0·10^-3^ the fingerprint peaks diminished rapidly and they were excluded as not suitable for identification.

Figure S4


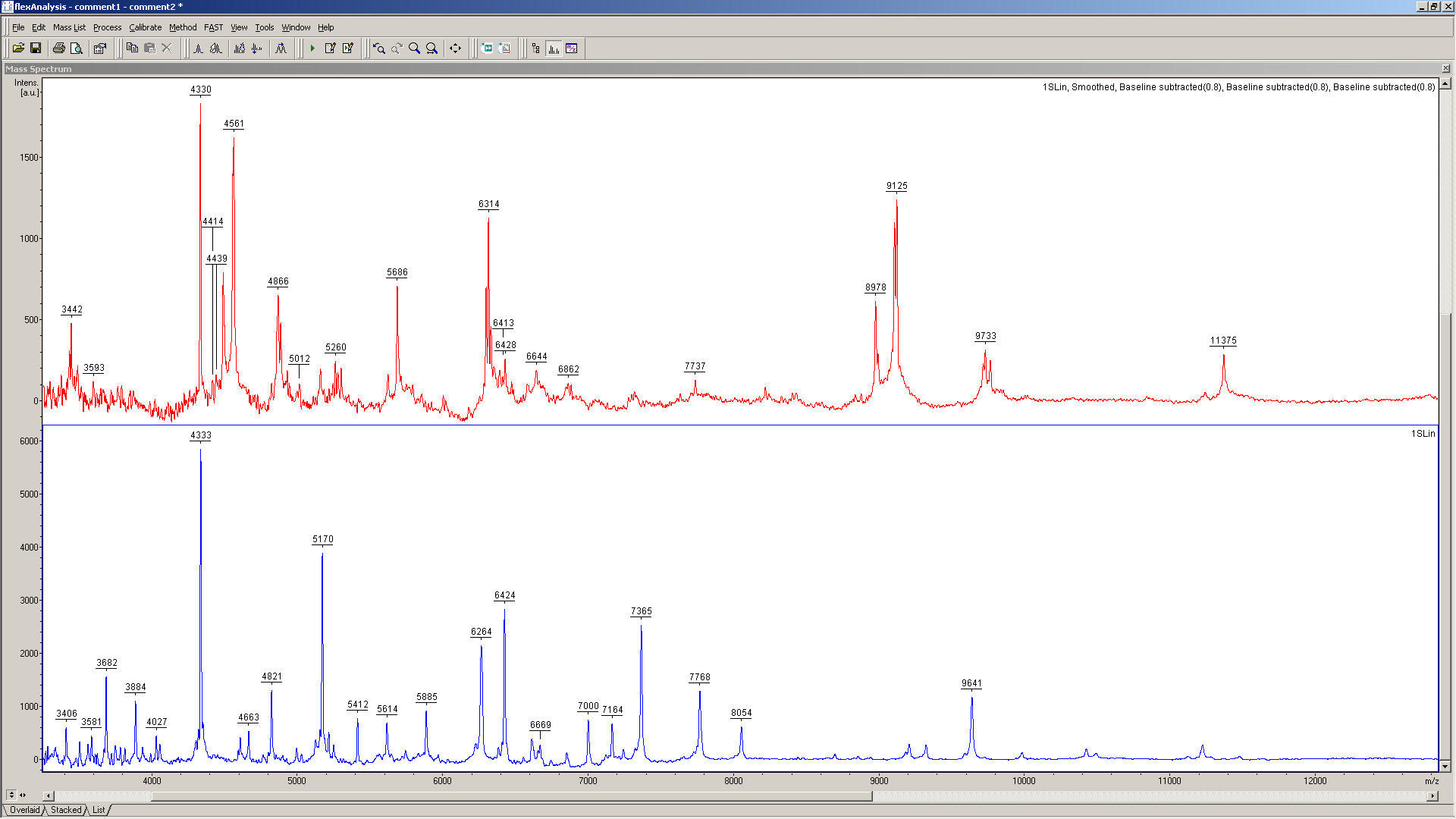


Figure S4 - Comparison of the MALDI-MS spectra H_2_O_2_ inactivated (red) and native (blue) *Bacillus anthracis*. Method protocol follows below.

AutoFlex MALDI-TOF MS protocol of inactivated bacteria

The results of DF-IEF fractionation were evaluated in a second laboratory using the MALDI-TOF MS analysis (AutoFlex, Bruker Daltonics) for routine identification of native bacteria. Glycerol containing samples were centrifuged at 10 000 g for 20 minutes and the pellets were washed with 2 mL of physiologic solution, vortexed and centrifuged again. The washing process was repeated twice. The DF‑IEF fractions were centrifuged at 10 000 g for 20 min and 0.7 µL of the supernatant was carefully taken at the bottom of the micro-test tube right above the pellet of cells and deposited on the MALDI sample plate (MTP 384 ground steel, Bruker) in a form of thin film so that at least 5 adjacent spots were covered. A culture of *E. coli* 3954 was also applied to the platform in the same way for calibration of the system. All sampled spots were then coated with the matrix solution ca 1.5 µL per spot (5 mg·mL^-1^ of α-cyano-4-hydroxycinnamic acid in 50% ACN and 2.5% TFA) and left at room temperature until the solvent evaporated and dried. After transfer of the platform to the AutoFlex system, the MS of each spot were acquired. For each spot, at least 300 individual spectra are accumulated to increase the signal-to-noise ratio, with an average resolution of at least 500 ppm. The data evaluation was performed using MALDI BioTyper ® system comparing the measured MS profiles with reference spectra from the MALDI Biotyper ® Security-Relevant (SR) Library.
